# Analysis of Exercise-Induced Periodic Breathing Using an Autoregressive Model and the Hilbert-Huang Transform

**DOI:** 10.1155/2018/4860204

**Published:** 2018-06-26

**Authors:** Tieh-Cheng Fu, Chaur-Chin Chen, Ching-Mao Chang, Hen-Hong Chang, Hsueh-Ting Chu

**Affiliations:** ^1^Department of Physical Medicine and Rehabilitation, Chang Gung Memorial Hospital, Keelung, Taiwan; ^2^Heart Failure Center, Department of Internal Medicine, Chang Gung Memorial Hospital, Keelung, Taiwan; ^3^College of Medicine, Chang Gung University, Tao-Yuan, Taiwan; ^4^Department of Computer Science, National Tsing Hua University, Hsinchu, Taiwan; ^5^Center for Traditional Medicine, Taipei Veterans General Hospital, Taipei, Taiwan; ^6^Faculty of Medicine, National Yang-Ming University, Taipei, Taiwan; ^7^School of Post-Baccalaureate Chinese Medicine, College of Chinese Medicine, Research Center for Chinese Medicine & Acupuncture, China Medical University, Taichung, Taiwan; ^8^Department of Chinese Medicine, China Medical University Hospital, Taichung, Taiwan; ^9^Department of Computer Science and Information Engineering, Asia University, Taichung, Taiwan; ^10^Department of Department of Medical Research, China Medical University Hospital, China Medical University, Taichung, Taiwan

## Abstract

Evaluation of exercise-induced periodic breathing (PB) in cardiopulmonary exercise testing (CPET) is one of important diagnostic evidences to judge the prognosis of chronic heart failure cases. In this study, we propose a method for the quantitative analysis of measured ventilation signals from an exercise test. We used an autoregressive (AR) model to filter the breath-by-breath measurements of ventilation from exercise tests. Then, the signals before reaching the most ventilation were decomposed into intrinsic mode functions (IMF) by using the Hilbert-Huang transform (HHT). An IMF represents a simple oscillatory pattern which catches a part of original ventilation signal in different frequency band. For each component of IMF, we computed the number of peaks as the feature of its oscillatory pattern denoted by Δ_*i*_. In our experiment, 61 chronic heart failure patients with or without PB pattern were studied. The computed peaks of the third and fourth IMF components, Δ_3_ and Δ_4_, were statistically significant for the two groups (both* p values* < 0.02). In summary, our study shows a close link between the HHT analysis and level of intrinsic energy for pulmonary ventilation. The third and fourth IMF components are highly potential to indicate the prognosis of chronic heart failure.

## 1. Introduction

**Figure 1:**
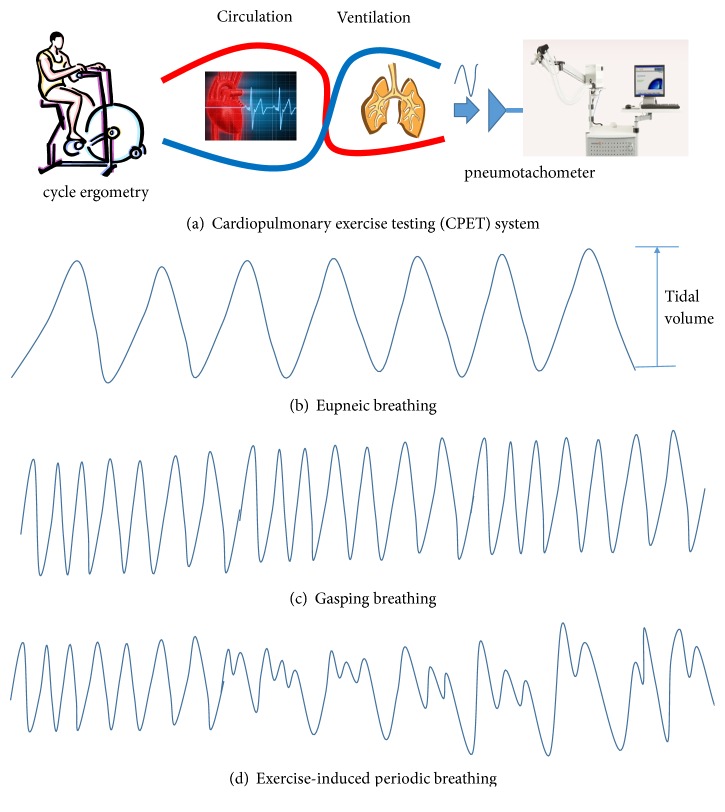
**A cardiopulmonary exercise testing (CPET) system and breathing patterns.** (a) A CPET machine is comprised of a cycle ergometer and pneumotachometer. During the test, the bicycle workload for the patient is increased until maximal exertion is reached. (b–d) Breathing patterns include eupneic, gasping, and periodic breathing (PB). Tidal volume is the volume of air exchange between inhalation and exhalation.

The rehabilitation of patients with chronic heart failure (CHF) is a slow process, and sometimes, good progress is difficult to obtain for some patients. Exercise-induced periodic breathing (EPB) was found to be an important evidence of poor prognosis [1–6]. Therefore, physiatrists commonly check exercise breathing patterns of patients with CHF by using cardiopulmonary exercise testing (CPET; Figure 1(a)) to guide the pharmacological and nonpharmacological treatments for these patients. CPET involves measurements of ventilation (VE) respiratory oxygen uptake (VO_2_) and carbon dioxide production (VCO_2_) during a symptom-limited exercise test [7]. On increasing the bicycle workload during a CPET test, the respiratory exchange rate and tidal volume increase simultaneously. For more respiratory exchanges, a periodic breathing (PB) pattern might occur in some patients with CHF. PB (Figure 1(d)), first described in the 1970s [8], is a phenomenon of abnormal hyperventilation that alternates apneas and hypopneas. In this study, we investigated the difference in ventilation signals between PB and non-PB patients [9].

From the analysis of cardiopulmonary exercise testing (CPET) measurements, two significant indicators have been studied in the literature, namely, peak VO_2_ and VE/VCO_2_ slope. Peak oxygen consumption (peak VO_2_) was considered the gold standard assessment parameter of prognosis in CHF [10, 11]. Then, the ratio of ventilation-to-carbon dioxide production (VE/VCO_2_ slope) was also studied later with the same importance as peak VO_2_ [12, 13]. More recently, the quantification of PB patterns was investigated in both the spatial and frequency domains [3–5]. Here, we endeavored to link CPET measurements and the quantification of PB patterns by using the Hilbert-Huang transform (HHT) [14]. The Hilbert-Huang transform has been applied in many biomedical analyses [15], including blood pressure [16, 17], nasal flow [18], and electroencephalography [19, 20]. To apply the Hilbert-Huang transform on the analysis of ventilation measurements, we propose two important steps of preprocessing. In the first step, we examine the ventilation measurements from CPET tests. Some ventilation measurements are noisy and aberrant when the testing patient is gasping. The occurrence of such aberrant signals is caused by the limitation of a CPET system. The ventilation VE values are obtained from breath-by-breath calculation of gas exchange at the mouth. A nonrebreathing valve is connected to a mouthpiece to prevent mixing of inspired and expired air. Thus, one irregular gasping exhalation may be recorded as two or more breaths. As a result, we filter out those aberrant measurements.

Moreover, not all measurements from entire CPET tests were used in our analysis. To determine the meaningful difference in cardiopulmonary response between the PB and non-PB patients, we only selected the period before the patient's ventilation reached the maximal volume. The common respiratory rate for an adult at rest is 12–20 breaths per minute, which will increase up to 30–50 breaths per minute during exercise testing. Thus, PB patterns are most likely to appear around the peak respiratory volume. Therefore, 200 ventilation measurements, that is, a period of 4–6 minutes before the peak volume, were used in the analysis.

## 2. Materials and Methods

### 2.1. Breath-by-Breath Ventilation Signals for 61 Patients with Chronic Heart Failure

Exercise ventilation signals were recorded from CHF patients who received rehabilitation at the Chang Gung Memorial Hospital-Keelung Branch in Taiwan. All the subjects were studied in accordance with a protocol previously approved by the local ethics committee and registered at the ClinicalTrials.gov website with ID No. NCT01053091. The respiratory signals were acquired using a pneumotachometer connected to a mask and analyzed using the machine MasterScreen CPX Metabolic Cart. In common cases, the signals, including VO_2_ and VCO_2_, are output per 30 seconds, although they are measured breath by breath. More information about the collected CPET data can be found in Fu et al. (2017). For this study, we output the original breath-by-breath signals of ventilation instead. The total measurement period was 10–15 minutes. We obtained 61 deidentified ventilation samples marked as PB (n = 20) or non-PB (n = 41) by physiatrists.

### 2.2. Filtering of Ventilation by an Autoregressive Model

**Figure 2:**
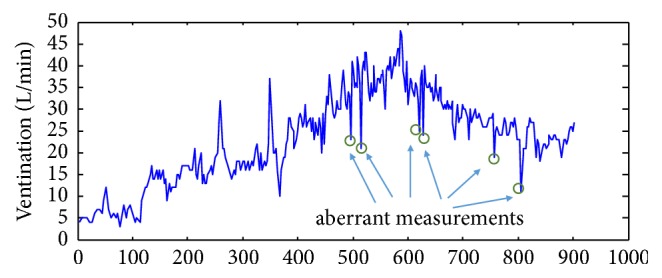
**Measured signals of exercise breath-by-breath ventilation.** Very low measurements usually occur when the patient is gasping. In this study, when the ventilation volume was high, these exceptional measurements were observed as noises.

Many observations of biosignal series exhibit serial autocorrelation and can be modelled with autoregressive (AR) models. Garde* et al.* showed that ventilation signals can also be fitted by AR models [18]. They used the coefficients of AR models to characterize the respiratory pattern of PB or non-PB patients. However, the average ventilation measurements per minute were adapted in their study. In our study, we analyzed breath-by-breath signals and applied the AR model method to fit the curve of exercise ventilation as shown in Figure 2.

The AR models predict* y*_t_ as a function of past observations, *y*_*t*−1_, *y*_*t*−2_,…, *y*_*t*−*p*_. The form of the AR model is

(1) $$\begin{array}{c} \bar{y_{t}}=c+\phi_{1}y_{t-1}+\cdots +\phi_{p}y_{t-p}, \end{array}$$

where* p* is the degree of the AR model and denoted by AR(*p*) and $\bar{y_{t}}$ is the predicted term.

For the analysis of PB or non-PB exercise ventilation, 200 serial measurements before the largest ventilation volume were chosen, and an AR(6) model is applied to the series. By using the equation to fit the exercise ventilations, we filtered out the observed measurement of ventilation* y*_t_ if $y_{t}<0.8\bar{y_{t}}$. This filtered series of ventilation signals is called “the most exhausted exercise ventilations (MEE-Ve)” in this paper.

### 2.3. Decomposition of the Chosen Ventilation Signals by the Hilbert-Huang Transform

The Hilbert-Huang transform (HHT) is a signal decomposition method developed by Norden E. Huang in the 1990s [14]. By using this processing method, biosignals are decomposed into a set of IMFs by an empirical mode decomposition (EMD) process. The instantaneous frequencies and amplitudes of all IMFs can be used to identify embedded signal structures.

The HHT representation of series* X(t)* is

(2) $$\begin{array}{c} X\left(\;t\;\right)=R\overset{n}{\underset{j=1}{\sum }}a_{j}\left(\;t\;\right)e^{i\theta_{j}\left(t\right)}=R\overset{n}{\underset{j=1}{\sum }}\left[\;C_{j}\left(\;t\;\right)+iY_{j}\left(\;t\;\right)\;\right], \end{array}$$

where* C*_j_(*t*) and* Y*_j_(*t*) are, respectively, the* j*-th IMF component of* X*(*t*).

**Figure 3:**
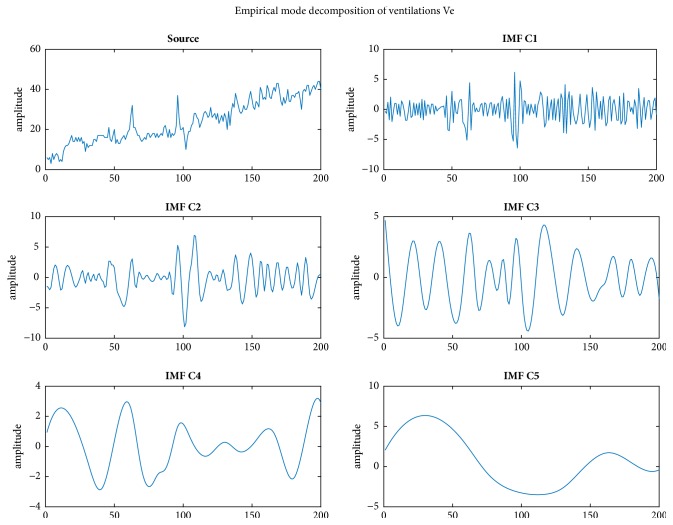
**An example of empirical mode decomposition of most exhausted exercise ventilations (MEE-Ve).** The MEE-Ve signals are *k* ventilations before the peak volume. The number *k* is 200 in this paper. The illustration is from the analysis of the patient ID: pb0001 which was judged as a periodic breathing (PB) case. All results of the EMD analysis for the PB or non-PB cases can be found at the Github repository (https://github.com/htchu/EpbAnalysis).

To obtain IMFs, EMD [14], which is an iterative process that output a set of signal components called IMFs, is performed.  Figure 3 shows an example of decomposed IMFs for a series of MEE-Ve. The original signals are decomposed into the components IMF_1_, IMF_2_,…, IMF_5_. Different IMF components may imply particular factors. We calculated the peaks of the oscillations in each IMF with MATLAB's “mspeaks” function [21]. The estimated peaks of the IMF components of IMF_*i*_ are denoted as Δ_*i*_ to compare the PB and non-PB samples.

### 2.4. Statistical Analyses

Student's t-tests were used to identify statistically significant differences between two groups of features of PB and non-PB samples.

## 3. Results

### 3.1. The Computation of IMFs of Most Exhausted Exercise Ventilations (MEE-Ve) for PB and Non-PB Patients

**Figure 4:**
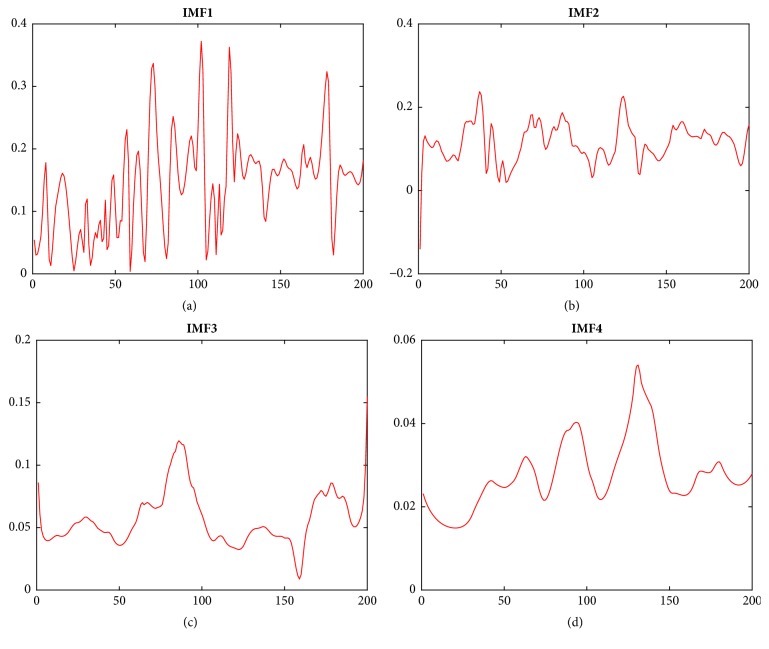
The corresponding instantaneous frequency of the decomposed IMF_1_-IMF_4_ (Figure 3).

The measurements of ventilation (VE) obtained from cardiopulmonary exercise testing (CPET) were analyzed using the proposed method. The programs were written in MATLAB. We analyzed the extracted exercise ventilations in this section by using HHT for 20 patients with or without PB as judged by physiatrists. The empirical mode decomposition (EMD) process is applied to the VE data and several IMFs are extracted.  Figure 3 depicts the HHT decomposition result for IMF1-IMF5. In addition, we show the corresponding instantaneous frequency of the decomposed IMF_1_-IMF_4_ in Figure 4. All figures of HHT decomposition results for the 61 patients are available at our Github repository (https://github.com/htchu/EpbAnalysis).

### 3.2. Numbers of Peaks as the Features of IMFs

**Figure 5:**
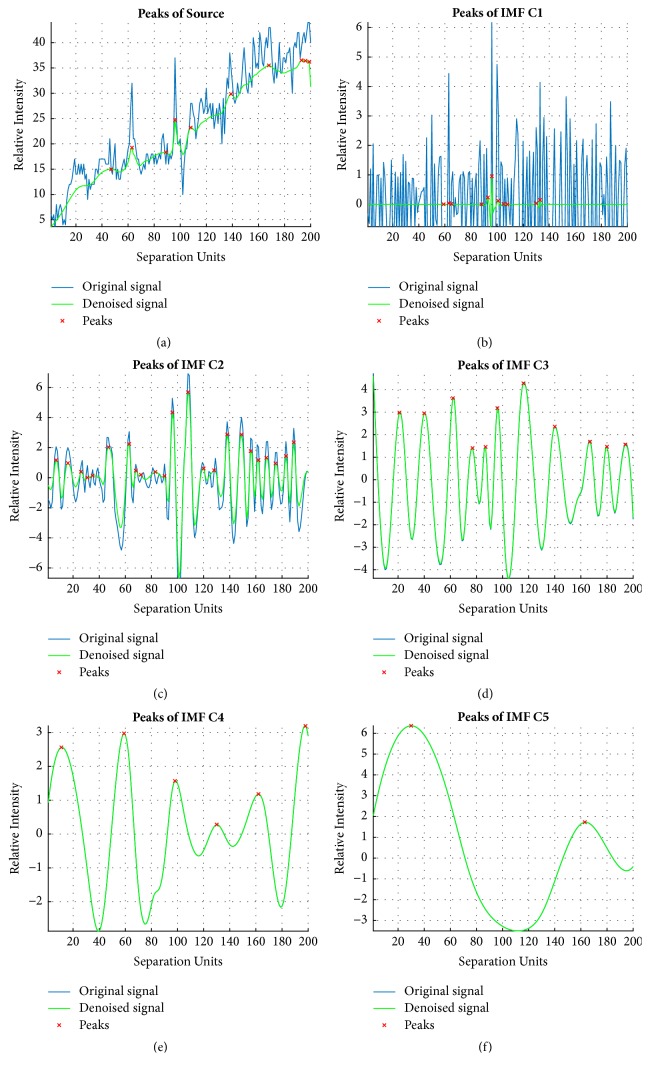
Peak Computations of IMFs (Figure 3).

We used MATLAB's “mspeaks” function to perform the peak fitting of IMFs and the source ventilations.  Figure 5 illustrates the computed locations of peaks for the same experimental data in Figure 3. Obviously, the peak fittings for the source ventilations and the first IMF (Figures 5(a) and 5(b)) are not as good as the peak fittings for the other IMFs (Figures 5(c)–5(f)).  Table 1 lists the numbers of computed peaks of IMF_1_-IMF_5_ for first 20 patients (10 PBs and 10 non-PBs). Supplemental Table 1 provides all of the computed peaks for the entire test dataset.

### 3.3. Statistical Significance Test of IMFs

**Figure 6:**
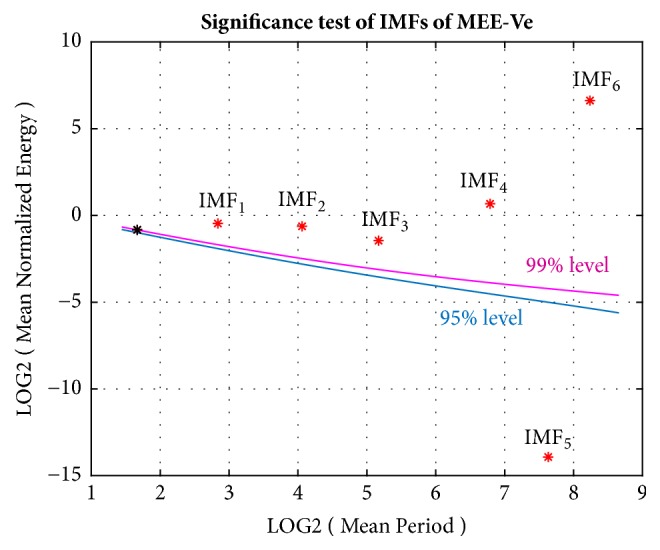
Statistical significance test for the decomposed IMFs. The IMF_5_ is below the 95% confidence limit and is therefore considered statistically insignificant.

The statistical significance test derived by Wu and Huang [22] is illustrated in Figure 6. The five extracted IMFs are shown along with the 95% and 99% confidence limits. All IMFs are above the 99-percentile confidence limit except for the IMF5. Therefore, only the IMF5 is not statistically significant from noise [22].

### 3.4. More Peaks of IMF_3_ and IMF_4_ for Better Prognosis of Chronic Heart Failure Cases

Student's t-tests were used to identify statistically significant differences between the two groups (PB and non-PB patients).  Table 2 lists the *p* values for the comparison between the two group for the computed peaks of IMFs. The *p* values for first two IMFs are greater than 0.1 such that the peak computations are not statistically significant for IMF_1_ and IMF_2_. By contrast, the *p* values for IMF_3_ and IMF_4_ are less than 0.02 such that the peak computations are statistically significant for IMF_3_ and IMF_4_.

## 4. Discussion and Conclusion

This paper conducted a new analysis on exercise ventilation signals to predict the prognosis of CHF patients. We defined MEE-Ve as a breath-by-breath ventilation measurement filtered using an AR model. We ran our correlation analysis through IMFs, extracted from the EMD process, and found that PB patterns were highly correlated to IMF_3_ and IMF_3_. To clarify the correlation, we introduced peak computation of IMF_3_ and IMF_4_ (Δ_3_, Δ_4_) as the feature of ventilation signals from cardiopulmonary exercise testing (CPET).

However, the effectiveness of the proposed method needs more clinical examinations in the future. Meanwhile, the range selection of exercise ventilations is another issue for more studies. We plan to examine this method with more cardiopulmonary tests.

## Figures and Tables

**Table 1:** Computed peaks Δ_*i*_ of IMFs by mspeaks.

PB or non-PB Patient	Δ_1_	Δ_2_	Δ_3_	Δ_4_	Δ_5_
PB-1	11	23	11	6	2

PB-2	2	28	17	7	3

PB-3	11	29	13	5	2

PB-4	9	30	13	7	3

PB-5	14	27	14	4	2

PB-6	8	25	13	5	2

PB-7	0	28	14	6	3

PB-8	21	25	16	8	3

PB-9	0	30	15	7	3

PB-10	3	23	9	5	2

nPB-1	7	26	16	6	3

nPB-2	7	29	15	8	2

nPB-3	8	28	16	9	4

nPB-4	2	24	14	7	3

nPB-5	4	28	17	10	5

nPB-6	0	30	17	7	5

nPB-7	15	26	15	6	2

nPB-8	8	27	15	7	3

nPB-9	9	26	16	8	4

nPB-10	2	31	17	8	3

**Table 2:** P values of Student's t-test for IMFs.

IMF component	Δ1	Δ2	Δ3	Δ4
*P value*	0.6330	0.1103	0.016	0.017

## Data Availability

The MATLAB programs and EPB data of this work are available at https://github.com/htchu/EpbAnalysis/.

## Supplementary Materials

Supplementary Materials Supplemental Table 1: computed peaks for the decomposed intrinsic mode functions (IMFs) from exercise ventilation signals of 61 chronic heart failure patients.
